# Properties and applications of [*n*]cumulenes

**DOI:** 10.3762/bjoc.22.110

**Published:** 2026-10-02

**Authors:** Julio César Hernández-Camacho, José Enrique Barquera-Lozada

**Affiliations:** 1 Instituto de Química, Universidad Nacional Autónoma de México, Circuito Exterior s/n, Ciudad Universitaria, 04510 Coyoacán, Ciudad de México, México

**Keywords:** electrohelicity, [*n*]cumulenes, parity, reverse bond-length alternation, terminal functionalizations

## Abstract

[*n*]Cumulenes are highly versatile sp-carbon scaffolds whose properties and potential applications are strongly dictated by their parity. This review provides an overview of the geometric and electronic structure, optical activity, conductance, and physicochemical behavior of [*n*]cumulenes containing three or more cumulated C=C double bonds, with particular emphasis on the contrast between even- and odd-membered systems. We also discuss their coordination chemistry and the related metalla[*n*]cumulenes, which exhibit notable similarities. Key features such as reverse bond-length alternation (BLA), the HOMO–LUMO gap, and electrohelicity are examined in the context of anti-ohmic behavior, chiroptical activity, and current-density patterns, and how these properties translate into design principles for molecular wires, spin filters, and single-molecule magnets (SMMs).

## Introduction

Carbyne is the idealized one-dimensional allotrope of carbon, composed entirely of sp-hybridized carbon atoms. This gives rise to two possible structures, one consisting of a sequence of alternating single and triple bonds (…–C≡C–C≡…), while the other is characterized by an infinite arrangement of equidistant double bonds (…=C=C=C=…). The latter arrangement is known as cumulenic carbyne, and it has been predicted that electron delocalization and conjugation along the infinite, rigid, and equidistant π system, confers metallic behavior to this structure. However, this arrangement is subject to Peierls distortion, so it experiences a transition to the alternating configuration of minimum energy, known as polyyne carbyne. This structure has a finite band gap and widens as the alternation between single and triple bonds increases, giving rise to semiconductor behavior [1–3].

While the existence of carbyne remains a contentious and controversial topic, this system is attractive for molecular electronics, since its versatility could be used for the design and construction of nanodevices with metallic, semiconducting or insulating properties. Furthermore, theoretical calculations have suggested that, under tension, carbyne undergoes a metal−insulator transition [4] and could be twice as tensile stiffness as carbon nanotubes and graphene, but also almost as flexible as polyaniline, with a persistence length of ≈14 nm [5]. This combination of mechanical and electronic properties has driven the synthesis and characterization of finite carbinoid structures, such as polyynes and [*n*]cumulenes (where “*n*” is the number of cumulated C=C double bonds). Although both families are defined as linear unsaturated hydrocarbons, polyynes are experimentally much more studied and described, since the longest polyyne synthesized to date contains 48 carbon atoms and 24 acetylene units [6]. Nevertheless, it is important to mention that there is another example with 68 carbon atoms and 34 acetylene units, but in this case the polyyne chain is stabilized with four threaded rotaxane macrocycles [7]. In contrast, the longest [*n*]cumulene synthesized and studied to date has only nine consecutive C=C double bonds in a chain of 10 carbon atoms [8–10] .

However, although polyynes are much more accessible synthetic targets, [*n*]cumulenes have had a relatively recent boom, thanks to their continuous arrangement of cumulated π bonds and their modifiability through specific terminal functionalizations. The latter allows precise control of their electronic and structural properties for the development of multiple possible applications. While it is true that experimental studies indicate that these molecules might offer exciting opportunities in the development of anti-ohmic wires [11], supercapacitors [12], organic field effect transistors (OFETs) [13–15], vibrational probes for live-cell SRS imaging [16–17], and nonlinear optical materials [18–19]; theoretical investigations suggest that they also could be promising for applications in spintronic devices [20], and single-molecule magnets (SMMs) [21]. Furthermore, although the reactivity of these compounds increases dramatically with increasing length, Januszewski's contributions have shown that it is possible to synthesize and study [*n*]cumulenes with more than five consecutive C=C double bonds [8–10]. Likewise, recent advances in cumulene chemistry established by Zhu's group have made it possible to successfully synthesize the first high-molecular-weight, stable, and solution-processable conjugated [5]cumulene polymers [22]. Nevertheless, there is an overwhelming number of publications on odd [*n*]cumulenes, while research on even ones remains scarce in comparison. This is partly because odd [*n*]cumulenes can be synthesized via homocouplings, self-condensations, or by incorporating acetylene units, which provides a wide variety of convenient synthetic routes, where the starting reagents are usually ketones [8–9,23]. But theoretical studies have shown that the differences in physicochemical properties between odd and even [*n*]cumulenes are significant, leading some authors to suggest that these two groups should be treated as separate classes of carbon chains [24]. Therefore, their relative energies may also contribute to the limited number of synthetized even [*n*]cumulenes, as it has been recently suggested [25].

Previous reviews have covered the structural, vibrational, and electronic properties of carbyne and finite carbinoid structures [1]. Synthetic routes to [*n*]cumulenes have also been discussed, and the physical and electronic structure of odd ones has been described using experimental UV–vis spectroscopy and X-ray crystallography data, also covering their reactivity [9]. Additionally, a complementary review also revisited these aspects and included the study of the electronic properties of odd [*n*]cumulenes, using experimental cyclic voltammetry data [26]. And more recently, the reactivity of [3]-, [4]-, and [5]cumulenes have been discussed in detail [27]. Nevertheless, here we review the properties and applications of [*n*]cumulenes, based primarily on theoretical investigations and some specific experimental results. The review is organized as follows. We begin by analyzing the electronic structure and structural properties of these compounds, then the electrohelicity phenomenon and optical properties; next, we address the conductance and the current density exhibited by these systems, moving on to their physicochemical properties and subsequently describe their coordination chemistry, ending with two brief chapters where potential additional properties are discussed.

## Review

### Electronic structure and structural properties

**Figure 1:**
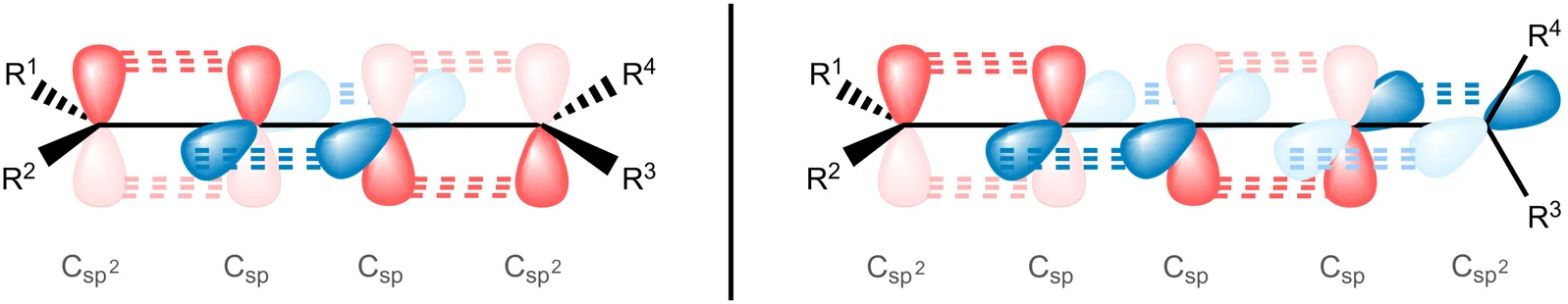
Simplified description of the electronic π structure for derivatives of [3]- and [4]cumulene.

[*n*]Cumulenes are unsaturated linear hydrocarbons [28] known as cumulated systems, since they are characterized by having “*n*” continuous C=C double bonds and “*n* + 1” carbon atoms [29]. The first member of this class of compounds, [3]cumulene (or buta-1,2,3-triene), exhibits three continuous or cumulated double bonds, which are formed between its two central carbon atoms, with sp hybridization, and its two terminal carbon atoms, with sp^2^ hybridization [30] (Figure 1).

**Figure 2:**
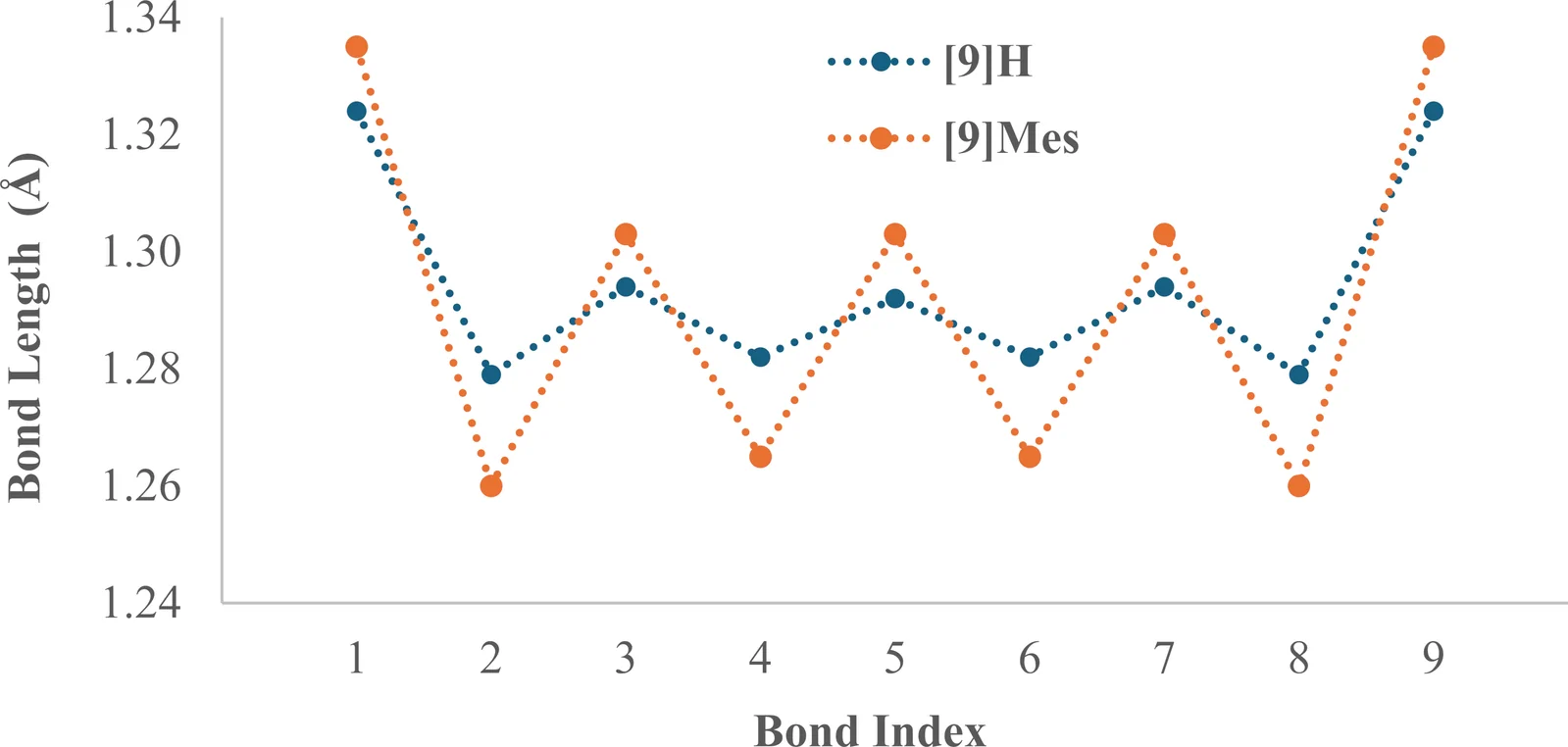
Comparison of C=C equilibrium bond lengths for [9]cumulenes with different terminal functionalizations. Theoretical values calculated with methylidene groups (=CH₂), at the B3LYP/6-31G* level of theory, are shown in blue [43], and experimental values with mesityl groups (Mes), determined via X-ray crystallography, are shown in orange [26].

Naturally, the consecutive presence of “*n*” C=C double bonds causes this family of compounds to exhibit *n* – 2 internal C_sp_=C_sp_ double bonds; these internal C atoms possess greater s-character, resulting in shorter bond lengths [26]. This factor, along with the cumulenic chain length, and its parity, as well as the electronic nature of the terminal functionalizations and their ability to optimally conjugate with the cumulenic chain, provoke the [*n*]cumulenes to exhibit bond-length alternation (BLA) [31–36]. This phenomenon is quite common in aromatic and conjugated systems, such as polyynes, polyenes, and polyacenes [37–40]. But [*n*]cumulenes exhibit reverse BLA in comparison, because they start with long and then short bonds [41–42], as seen in Figure 2.

**Figure 3:**
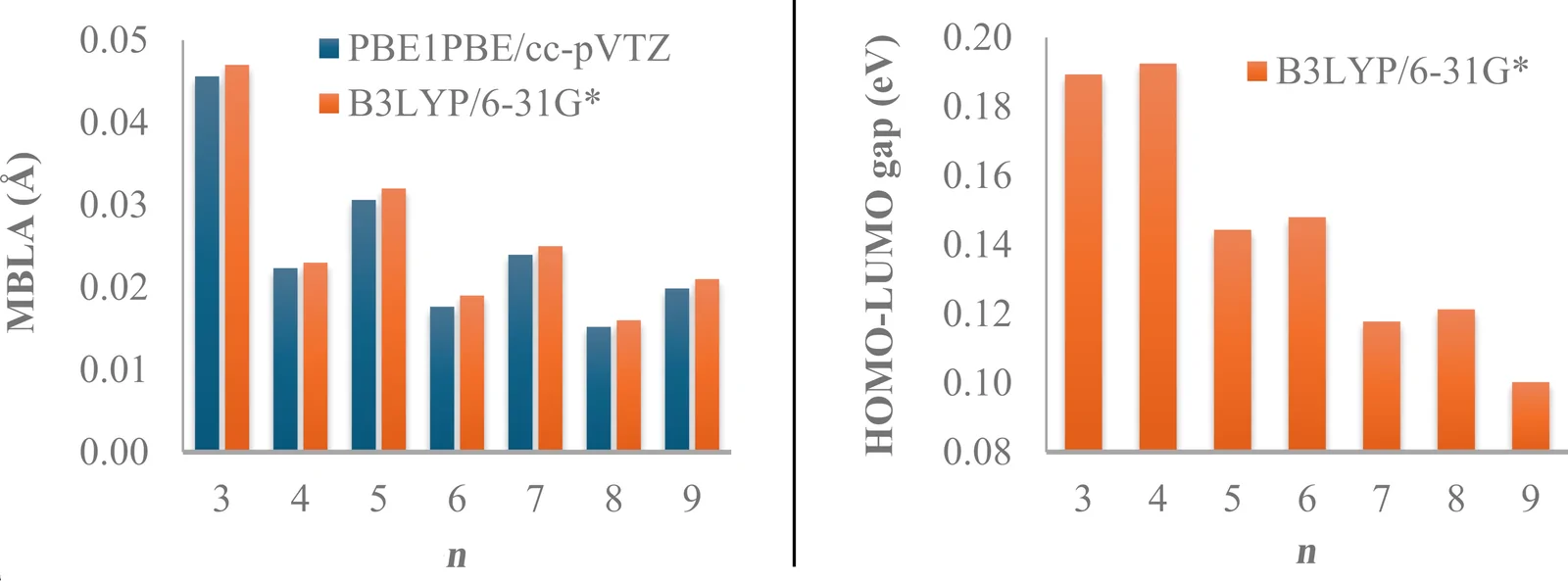
Comparison of the calculated mean bond-length alternations (MBLA) and HOMO–LUMO gaps of bis-functionalized [*n*]cumulenes with methylidene groups (=CH_2_). Theoretical values at the B3LYP/6-31G* level of theory are shown in orange [43] and theoretical values at the PBE1PBE/cc-pVTZ level of theory are shown in blue [38].

Originally, Januszewski and Tykwinski defined the BLA in [*n*]cumulenes as the difference between the lengths of the two central-most C=C double bonds in the cumulenic chain [9]. But, by averaging the difference between the lengths of all contiguous C=C double bonds in the cumulenic chain – to calculate an average BLA or MBLA, – the following trends have been observed:

**I. As the cumulenic chain increases, the MBLA values decrease (tending to an asymptotic limit) (** Figure 3 **).**

**Figure 4:**
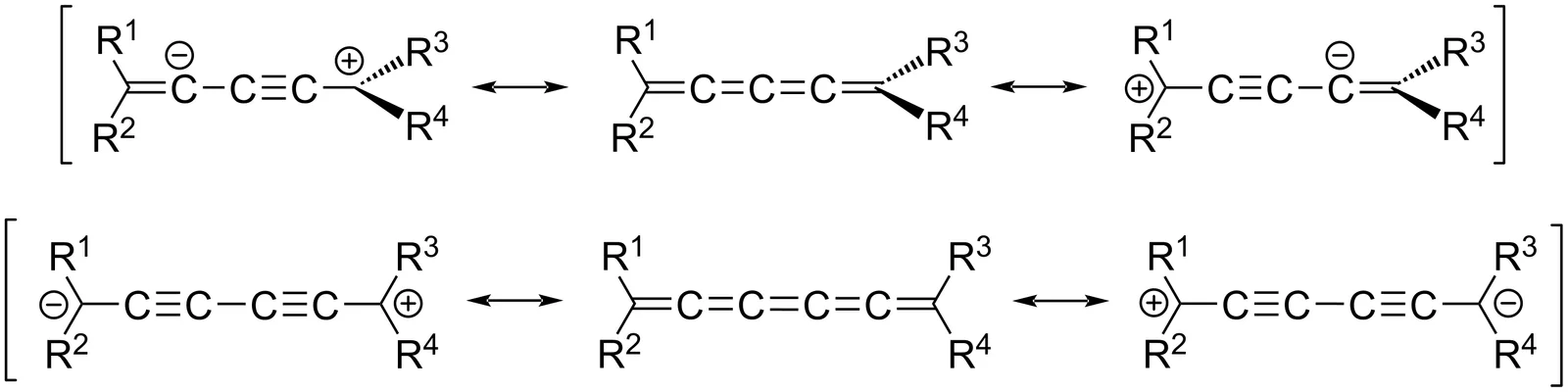
Canonical resonance structures for derivatives of [4]- and [5]cumulene [23].

This decrease of BLA and reach of an asymptotic limit is like other types of carbon wires and it can be falsely related to an increasing π-conjugation [1]. However, [*n*]cumulenes have a reverse BLA which has been explained with their resonance structures (Figure 4) or with their molecular orbitals [44]. These two models coincide that a large reverse BLA would increase the π-conjugation, which is contrary to the equalization that occur when the length of the chain is increased. The real reason for the reduction of BLA is the reduction of the terminal group effects toward the center of the chain. This effect can be seen in Figure 2, where the distances of the C_sp_=C_sp_ double bonds tend to equalize at the center of the chain; although this is more evident in [9]H, since the BLA of its most central (or most internal) pair of C=C double bonds is 0.010 Å, but it can decrease to 0.006 Å for *n* = 19, 27 and 39 [45] and can even be less than 0.005 Å for *n* = 99 [4].

**II. Even [** ***n*** **]cumulenes (** ***n*** **= 4, 6, and 8) exhibit smaller MBLA values compared to their odd homologues (** ***n*** **= 3, 5, 7, and 9).**

Naturally, this trend can only be appreciated through theoretical studies, since experimentally there are only four reported crystal structures of even [*n*]cumulenes and all of them correspond to [4]cumulenes [46–49], to date, not a single [8]cumulene has been synthesized, and the only [6]cumulene reported so far only exists in solution [50].

This trend has been explained through the resonance structures shown in Figure 4. For an odd number of continuous C=C double bonds, optimal delocalization exists, giving rise to zwitterionic (or diradical) resonance structures with alternating single and triple bonds. Nevertheless, when an even number of continuous C=C double bonds are present, the conjugation is never complete, one of the terminal bonds always retains its double character. This symmetry difference results in higher MBLA values for odd [*n*]cumulenes and also causes their BLA to be more reverse. But, these resonance structures also show that the BLA cannot be related to the degree of delocalization in even [*n*]cumulenes, because their two resonance structures cancel each other by symmetry, and consequently, the BLA would neither increase or decrease with larger or lesser conjugation.

The fact that BLA and electron delocalization are related phenomena is not surprising, since BLA also corresponds to one of the most popular geometric indices to estimate electron delocalization in aromatic and conjugated systems. If BLA tends to zero, it means that the system is highly delocalized [51–52]. But it is important to emphasize that this model only applies to aromatic and conjugated systems, because if we try to extend its use to [*n*]cumulenes, the BLA increases as the delocalization increases for odd ones, while for even ones there isn't direct correlation between BLA and the electron delocalization. Therefore, we consider that BLA should not be used as a geometric index to estimate electron delocalization in cumulated systems. On the other hand, beyond the BLA, to date no comparative study on electron delocalization in even and odd [*n*]cumulenes has been carried out, but other geometric indices have been used to quantify electron delocalization, specifically in 1,10-heterodisubstituted [9]cumulene derivatives, such that, through the Harmonic Oscillator Model of Aromaticity (HOMA) [53].

**III. As the cumulenic chain increases, the HOMO–LUMO gaps decrease (**Figure 3**)** [45,54]**.**

Although in this case this behaviour is only observed through the results with bis-functionalized [*n*]cumulenes with methylidene groups (=CH_2_), in general the trend is preserved regardless of the electronic nature of the terminal functionalizations and this phenomenon is also quite common in aromatic and conjugated systems, such as polyynes, polyenes, and polyacenes; as their repeating units increase, delocalization increases, and, consequently, their HOMO–LUMO gaps decrease [41–42]. However, the gap is always smaller in [*n*]cumulenes. In polyenes and polyynes, the HOMO−LUMO gap is larger because the HOMO reduces its energy due to the large BLA (Table 1, entries a and b), while the LUMO increases its energy due to the same structural cause [42]. In [*n*]cumulenes with the same number of carbon atoms, the HOMO and LUMO have a similar shape, but the BLA is significantly smaller. Increasing the BLA in [*n*]cumulenes would have a high-energy cost because it would increase the energy of the HOMO−1, which is localized on bonds contiguous to the HOMO (Table 1, entries c and d), which is not the case for polyenes and polyynes.

**Table 1:** Optimized structures and FMOs (HOMO−1 to LUMO) of a)1,1,4,4-tetramethyl-[2]polyene (2,5-dimethylhexa-2,4-diene), b) 1,4-dimethyl-[2]polyyne (hexa-2,4-diyne), c) 1,1,4,4-tetramethyl-[3]cumulene and d) 1,1,5,5-tetramethyl-[4]cumulene, computed at the M06-2x/Def2-TZVP level of theory.

Entry		HOMO−1	HOMO	LUMO

a)	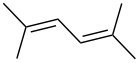 1,1,4,4-tetramethyl-[2]polyene	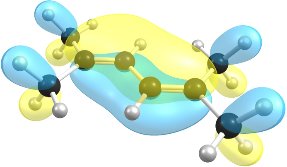	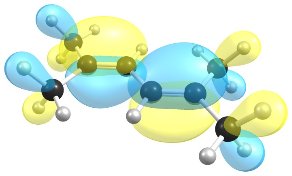	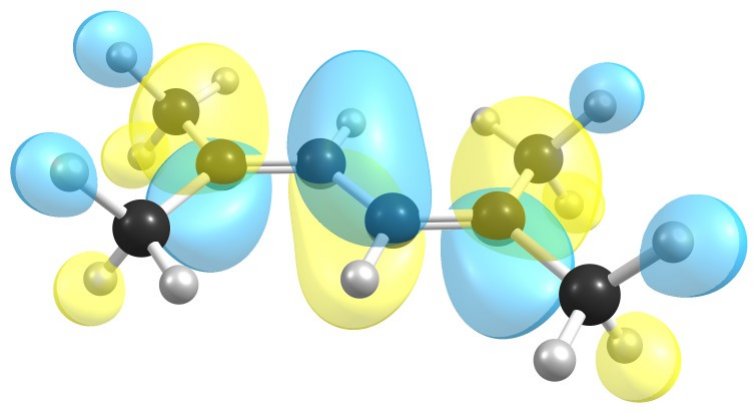
b)	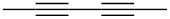 1,4-dimethyl-[2]polyene	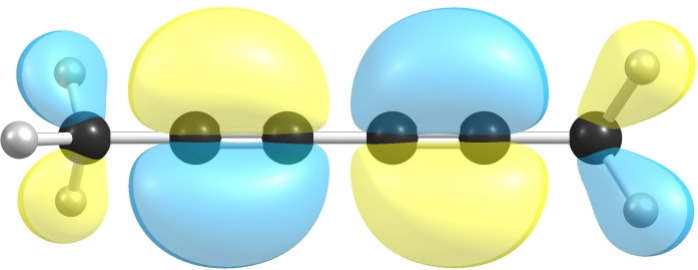	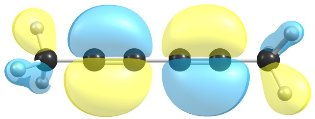	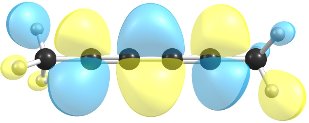
c)	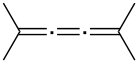 1,1,4,4-tetramethyl-[3]cumulene	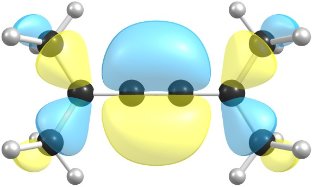	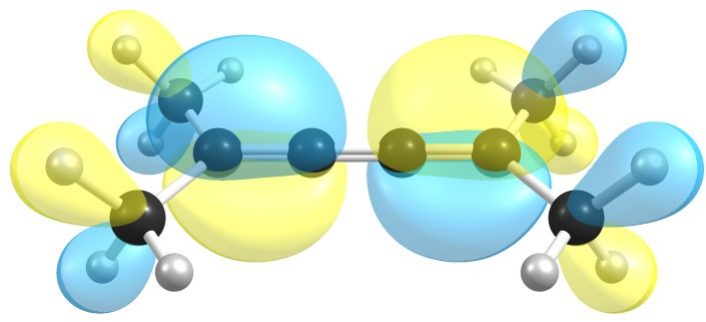	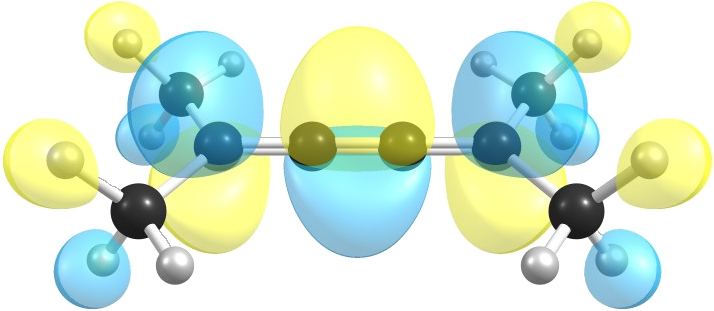
d)	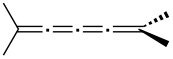 1,1,5,5-tetramethyl-[4]cumulene	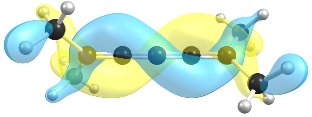 π*_P_*	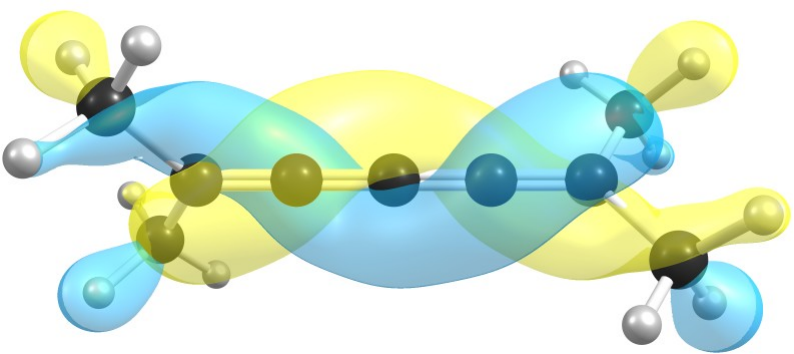 π*_M_*	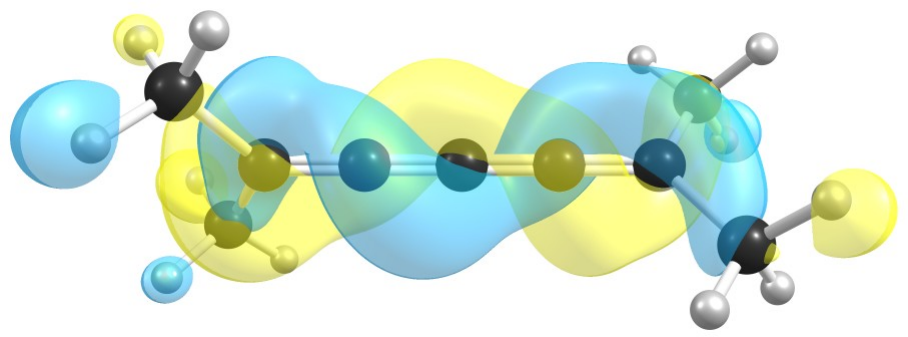 π_P_*

*For the case of d), the symbols π_P_ and π_M_ indicate that its helical π-FMOs have a specific helicity (or rotation). (*P*) (“plus”) represents a counterclockwise rotation (or a right-handed π-helix) and (*M*) (“minus”) represents a clockwise rotation (or a left-handed π-helix).

**IV. Odd [** ***n*** **]cumulenes (** ***n*** **= 3, 5, 7, and 9) exhibit smaller HOMO–LUMO gap values compared to their even homologues (** ***n*** **= 4, 6, and 8).**

This difference has been attributed to the coplanarity of terminal functionalizations in odd [*n*]cumulenes (Figure 1), which allows complete end-to-end electron conjugation and delocalization, thereby reducing the HOMO−LUMO gap. In contrast, in even [*n*]cumulenes the terminal functionalizations are perpendicular, which limits the electronic communication across the π-system of the molecule and hence results in a wider HOMO−LUMO gap [55–56]. However, this HOMO−LUMO gap differences are also present in bis-functionalized [*n*]cumulenes with methylidene groups (=CH₂), which cannot conjugate with the chain. The shorter gap in odd [*n*]cumulenes can be better understood with a Hückel secular determinant. In [*n*]cumulenes, this determinant can be constructed with two sets of π orbitals perpendicular to each other. Therefore, the determinant is block-partitioned, and the HOMO−LUMO gap depends on the size of the largest block. In even [*n*]cumulenes, the two sets of conjugated π orbitals are of the same size; thus, the blocks in the determinant are equal (*n* × *n*) [44]. But for odd [*n*]cumulenes, the two sets of atomic π orbitals are not equal: one is (*n* + 1) × (*n* + 1) and the other is (*n* − 1) × (*n* − 1). Consequently, the former determines the size of the gap. Accordingly, the HOMO−LUMO gap calculated at Hückel level for a [2*m*]cumulene (*m* is an integer) is the same as for a [2*m* − 1]cumulene. Therefore, the HOMO−LUMO gap is determined by the maximum number of overlapping π orbitals within the chain. This trend persists at higher levels of theory and can be enhanced if the carbon chain conjugates with the terminal groups.

**Figure 5:**
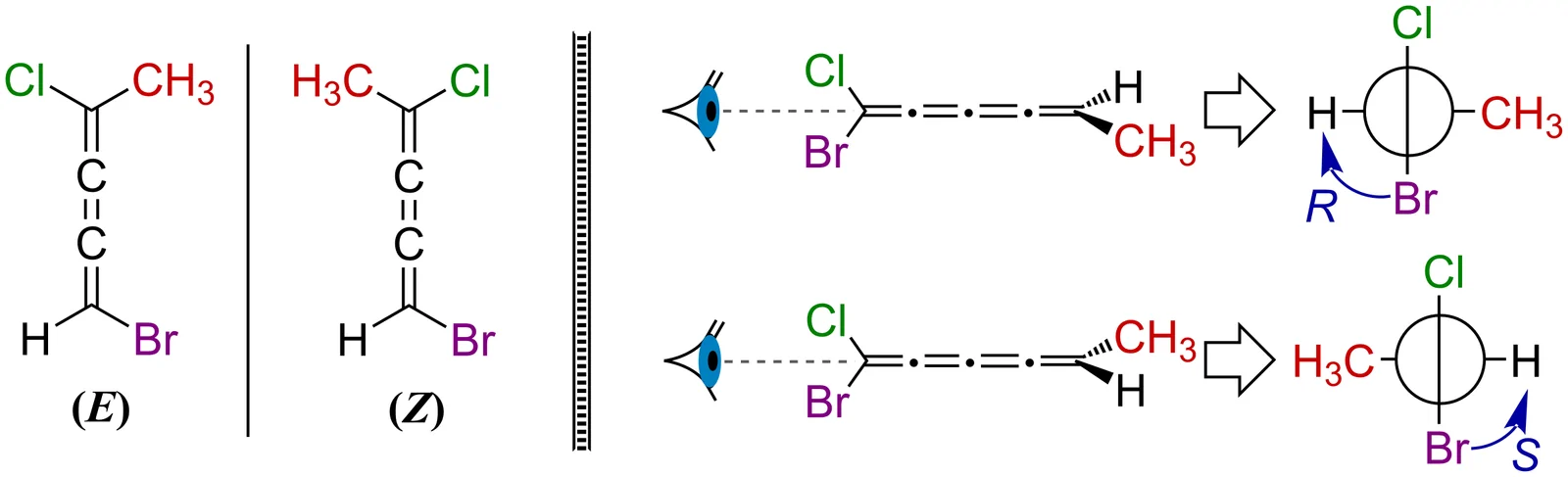
Stereochemical properties for even and odd [*n*]cumulenes.

Now, based on the above, [*n*]cumulenes are divided into two subclasses distinguished by the parity of the number of cumulated C=C double bonds they possess [49], which causes these compounds to also exhibit distinct stereochemical properties. On the one hand, the orientation of the terminal functionalizations in odd [*n*]cumulenes gives rise to planar structures, with *D*_2_*_h_* symmetry and allows geometric isomerism in substituted derivatives, as occurs in alkenes [30] (Figure 5). Consequently, *D*_2_*_h_* symmetry also generates two sets of non-degenerate rectilinear π frontier molecular orbitals (FMOs) (Table 1, entry c), which extend from end-to-end along the entire molecule. This is magnified with the presence of arylic terminal functionalizations or some other functional group that promotes the rigidity of these systems, maximizing the end-to-end conjugation and delocalization.

On the other hand, terminal functionalizations in even [*n*]cumulenes lie on mutually perpendicular planes, giving rise to a *D*_2_*_d_* symmetry and allowing axial chirality when the symmetry is reduced by the terminal functional groups to *C*_2_ or lower [44] (Figure 5). In this reduction of symmetry, the degenerate pair of rectilinear π-FMOs (π_x_ and π_y_) mix and form a pair of *quasi*-degenerate helical π-FMOs (HOMO−1 and HOMO, LUMO and LUMO+1, etc.), one of each helicity (*P* and *M*) [57] (Table 1, entry d). This phenomenon is relatively recent and has been termed "electrohelicity" [58]. This concept will be discussed in detail in the next section.

### Electrohelicity and optical properties

The helical topology has led to even [*n*]cumulenes being considered coarctate Möbius systems. This is intrinsic to their electronic structure and can be seen in Figure 1; the 2p atomic orbitals at the ends of the cumulenic chain are oriented perpendicularly, which naturally generates a sign change in the overlap sequence. This sign change is a characteristic feature of cyclic Möbius systems, but in this case, it occurs in a linear structure, where the rectilinear π molecular orbitals (MOs) of two orthogonal sets (π_x_ and π_y_) mix, and this phenomenon is called "coarctation" because the two π systems are fused or overlapped on the same atoms [44].

It is worth mentioning that *D*_2_*_d_* symmetry can grant degenerate pairs of FMOs, both π helical and π rectilinear, since it only depends on the algorithm for choosing the symmetry of the initial estimation orbital, used in self-consistent-field calculations [59–60]. For this reason, Table 1 (entry d) shows a HOMO resulting from a *D*_2_*_d_* symmetry, with (*M*)-helicity and its degenerate pair, the HOMO-1, has (*P*)-helicity. Nevertheless, the symmetry reduction *D*_2_*_d_* → *C*_2_, *D*_2_ or *C*_1_ leads only to the formation of pairs of π helical FMOs, in principle *quasi*-degenerate, but that depends on the enantiomerism of the terminal functionalizations. Through theoretical studies, the substitution pattern and the functional groups, which allow widening the energy gap that separates the HOMO and HOMO−1, in [4]cumulene derivatives have been investigated. One work proposes the tetrasubstitution with electron-withdrawing and electron-donating π groups at opposite ends of the molecule, reducing the symmetry *D*_2_*_d_* → *C*_1_, forming a *push–pull* system that produces a gap of 0.42 eV in one example [60]. However, the largest gap to date is obtained by reducing the symmetry *D*_2_*_d_* → *C*_2_ with pyramidal electron-donating π groups, achieving a gap of up to 0.461 eV [61]. Although this approach has also been used with electron-donating σ groups, capable of hyperconjugating with the helical π system, achieving a gap of 0.11 eV [57]. While it is true that MOs are not experimentally observable, this topic is of considerable interest because if the simultaneous presence of both helicities in the electronic structure is eliminated, even [*n*]cumulenes would become potentially useful for optical detection, photoanalysis, enantioselective transformations, and screw electromagnetism [60].

Now then, at this point, it can be considered that electrohelicity and optical activity are mutually correlated and exclusive properties of even [*n*]cumulenes. Nevertheless, through theoretical studies, it has been determined that axial torsion reduces the symmetry of unsubstituted [*n*]cumulenes to *D*_2_ if the dihedral angle (φ) formed between the methylidene groups (=CH_2_) is 0° < φ < 90°, giving rise to chiral molecules with electrohelicity [44] but with different responses to optical rotation (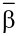). For even [*n*]cumulenes, 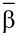 is maximum when φ = 10°, but for odd ones, 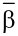 is maximum when 60° ≤ φ ≤ 70° [62]. It is worth mentioning that axial torsion also eliminates the *quasi*-degeneracy of helical π-FMOs in even [*n*]cumulenes and increases their optical activity, because if φ ≠ 90°, the transitions become optically active [63]. On the other hand, through theoretical studies, it has also been determined that odd [*n*]cumulenes can exhibit chirality without electrohelicity. This has been confirmed through 1,4-disubstitution in [3]cumulene derivatives, reducing their symmetry *D*_2_*_h_* → *C*_2_, giving rise to specific rotations similar to those of equivalently 1,5-disubstituted [4]cumulenes [57]. Therefore, electrohelicity and optical activity aren't mutually correlated properties, and they aren't exclusive to even [*n*]cumulenes.

To date, most recent theoretical studies have focused on quantifying helical morphology, using the trajectories of the stress tensor Tσ(s) [29], the mean absolute deviation (MAD) [64] or the correlation quantity between a hypothetical perfect helix and the exact feature of the π-system under consideration (HEL) [62]. But electrohelicity in even [*n*]cumulenes is more than just helical orbitals, it is a phenomenon that generates completely delocalized FMOs along the entire molecule [61]. Therefore, it is a property that could also be useful for molecular electronics.

### Conductance and current density

Molecular electronics is an area of chemistry oriented towards materials science and corresponds to the study and application of organic molecules, which act as transistors, rectifiers, switches, wires and other components for the manufacture of electronic nanodevices [65]. Molecular wires are the fundamental building blocks for that; in general, highly conjugated and delocalized molecules have been investigated [66], but specifically, they must meet at least four requirements:

- Rigidity, since fluxionality modifies conductance [53,67].
- Optimal electrode–molecule–electrode coupling [68].
- FMOs completely delocalized along the entire molecule, providing an efficient conduction route for electron transport [69].
- Length-independent conductance or, better yet, anti-ohmic behavior; that is, conductance increases with length [41].

In this sense, [*n*]cumulenes show a priori all the characteristics required to be molecular wires:

- They are rigid systems, which minimizes conformational changes at junction sites [53,67].
- The gradual reduction of their HOMO–LUMO gap, as their cumulenic chain increases, consequently, increases their conductance. Therefore, they exhibit anti-ohmic behavior, as a direct consequence of their reverse BLA [42]. Although it has only been experimentally confirmed in odd [*n*]cumulenes [11].
- Arylic terminal functionalizations provide completely delocalized FMOs along the entire molecule and at the junction sites, giving optimal electrode-molecule-electrode coupling and providing an efficient conduction route for electron transport [41].

However, these characteristics are only at a general level, since specifically, many theoretical studies have concluded that only odd [*n*]cumulenes are promising prospects for molecular wires [70–73]. On the one hand, this is because their BLA is more reverse, which gives them a more anti-ohmic character. On the other hand, the destructive quantum interference (DQI) effect, due to the quasi-degeneracy of pairs of π-helical FMOs with opposite symmetries, results in negligible electron transmission [24]. Therefore, if the simultaneous presence of both helicities in the electronic structure can be eliminated, even [*n*]cumulenes could also be promising prospects for molecular wires. Currently, this possibility has only been evaluated theoretically, through 1,5-disubstituted [4]cumulenes with pyramidal electron-donating π groups, achieving an electron transmission similar to those of equivalently 1,6-disubstituted [5]cumulenes [61]. Although, it should also be noted that another recent theoretical approach has been through axial torsion, giving rise to diradicals [4]cumulenes without electrohelicity but with higher conductances than diradical [5]cumulenes with electrohelicity [24].

In this regard, it has recently been suggested that diradical character is a prerequisite for the design of extremely conductive anti-ohmic wires [74–75]. But some authors have concluded that very long odd [*n*]cumulenes (*n* ≥ 11) suffer spin symmetry breaking due to their pronounced diradical character, causing their HOMO–LUMO gap to increase as their cumulenic chain increases, giving a consequent decay in conductance [41]. Moreover, the diradical character can also lead to the obtaining of highly reactive and difficult-to-handle compounds. Therefore, a promising alternative to increase the anti-ohmic behavior in odd [*n*]cumulenes has been through the increase of its reverse BLA by electron-withdrawing and electron-donating π groups at opposite ends of the molecule, forming a *push–pull* system, which naturally induces the zwitterionic resonance structures shown in Figure 4 [42]. Specifically, this property is known as proacetylenicity and describes the intrinsic tendency of the central bond in *push–pull* [3]cumulenes to adopt an acetylene-like structure and reactivity. Compounds belonging to this definition are also called proacetylenic chromophores and are characterized by exhibiting tiny HOMO–LUMO gaps and exceptionally low rotational barriers. Consequently, proacetylenicity provides a powerful design principle for the development of low-energy gap organic materials and for the construction of molecular motors, respectively [76–77].

Although conductance is currently only a promising property for study in odd [*n*]cumulenes, the vector field that shows how this quantum electron flow is spatially distributed through the cumulenic structure is a promising property for study in even [*n*]cumulenes, since, as we will see, the current density consequently also exhibits distinctive patterns according to parity, as discussed below:

- In odd [*n*]cumulenes, the current density follows a nearly linear path through the π system, but in even ones, the current density flows circularly around each carbon atom in the cumulenic chain, giving rise to ring currents, where the direction is controlled by the helicity of the FMOs [24,61].
- In odd [*n*]cumulenes, the nodal plane is observed in the current density, exactly located in the molecular plane, while in even ones, the nodal plane is not observed in the current density [61,78].

Through theoretical studies, it has been determined that circular currents arise because FMOs have helically propagating nodes, and the gradient at these nodes creates a circular component in the current density. Moreover, the nodal plane is not observed in the current density due to the presence of pairs of quasi-degenerate FMOs; by symmetry, the nodal planes of these MOs are mirror images of each other, causing each MO to emphasize the current density in regions where its pair suppresses it [78].

On the other hand, theoretical investigations have demonstrated that odd [*n*]cumulenes can also exhibit circular currents by axial torsion, and the only difference with even ones is that these flow around the bonds, but they can also rotate clockwise and anti-clockwise, depending on the direction of torsion, and they persist even when the torsion angle is close to 0 and 180º [24].

Now then, the possible applications that may arise from the presence of circular currents are listed below:

1. **Magnetic control of conductance**. While it is true that conductance is negligible in even [*n*]cumulenes due to the DQI effect, the presence of a local unidirectional magnetic field, generated by circular currents, could reverse this [24]. But this hasn't been investigated further.
2. **Synthetic molecular motors**. Since the circular currents around an axis could drive the unidirectional rotation in even [*n*]cumulenes, thus making them function as a current-driven motor [24]. Nevertheless, this remains only a suggestion – as does the previous possible application – because there is still no further evidence to support it.
3. **Induction of substantial magnetic fields**. Recently, a theoretical study demonstrated that in single-molecule junctions, linear carbon chains with circular currents generated by helical π-systems could produce magnetic fields in the mT range. This was achieved in 1,5-diamino-[4]cumulene and 1,6-diamino-[5]cumulene with their end groups rotated to 60° by axial torsion. But when the bias window is gated closer to HOMO resonance, the small diameter of the unidirectional ring current around 1,5-diamino-[4]cumulene resulted in the generation of a magnetic field in the sub-tesla range with a maximum field strength of 0.15 T, approaching the fields of EPR magnets [79].

While all these applications are still at the theoretical or proof-of-concept stage, they provide possible experimental directions for promoting the synthesis and further exploration of even [*n*]cumulenes.

### Physical chemistry

**Figure 6:**
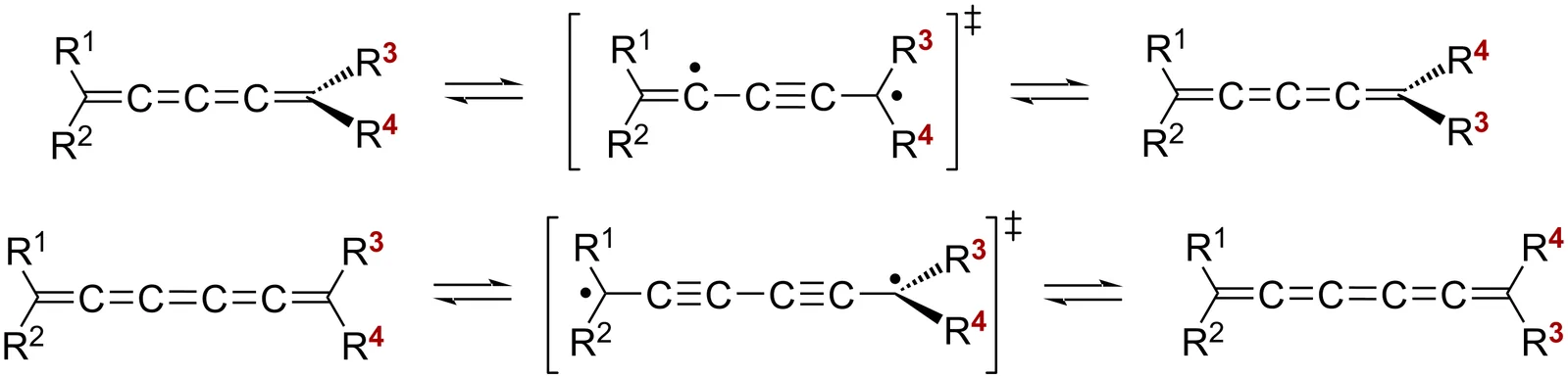
Internal rotation mechanism, in the absence of electric fields, for nonpolar derivatives of [4]- and [5]cumulene.

The diradical character of [*n*]cumulenes has also been proposed in theoretical studies of their rotational barriers. Figure 6 shows that the internal rotation mechanism occurs thanks to the presence of a diradical transition state; although, it could also be through a zwitterionic state, but most of the theoretical and experimental investigations have studied rotational barriers of non-polar [*n*]cumulenes [43,80–87]. To date, there is only one study on *push–pull* [3]cumulene rotational barriers [76]. Although, it has also been shown that the isomerization of non-polar [3]cumulenes occurs via a zwitterionic transition state when electric fields are applied [88].

In general, all theoretical and experimental studies have concluded that rotational barriers decrease as the cumulenic chain increases [43,80–87]; although, experimentally, the magnitude of this decrease has only been quantified in odd [*n*]cumulenes (Table 2), since to date, only one study related to [4]cumulenes has been carried out [89]. Nevertheless, it has recently been suggested that this decrease is because the geometric and electronic structure in the ground state begins to acquire similarities with the geometric and electronic structure in the transition state, as the cumulenic chain increases [80].

**Table 2:** Experimental values of rotational barriers (Δ*G*^≠^_rot_) of tetraaryl-[*n*]cumulenes. (*n* = 2 + *m*).

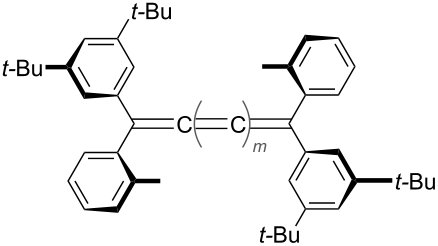	*n*	Δ*G*^≠^_rot_ (kcal/mol)

	3	>24.0
	5	19.1 ± 0.5^a^
	7	14.5 ± 0.4^a^
	9	10.9^a^/11.0^b^

Values were determined by VT ^1^H NMR, using ^a^CLSA and ^b^the approximation/estimation method [80].

On the other hand, much of the theoretical work on rotational barriers has been carried out on [*n*]cumulenes bis-functionalized with methylidene groups (=CH_2_). This has allowed to demonstrate that for extremely long [*n*]cumulenes (*n* → ∞), the rotational barriers tend to an asymptotic limit of 0.2 eV [84]. Moreover, it has also been shown that there is a linear correlation between the decrease in the HOMO–LUMO gap and the decrease in rotational barriers; while this is true for both even and odd [*n*]cumulenes, the correlation is slightly larger for even ones (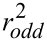
= 0.9976 and 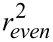 = 0.9996) [43].

Additionally, other physicochemical properties of these systems have also been studied; for example, the unimolecular dissociation from [3]- to [5]cumulene has been investigated and it has been discovered that it is based on the cleavage of C–H bonds, where the loss of atomic hydrogen is the dominant channel [90]. Proton affinities have also been calculated for chains from *n* = 3 to 20, which has allowed to determine that odd [*n*]cumulenes exhibit higher proton affinities than their even homologues, although this difference is reduced as the cumulenic chain increases [30], this indicates that odd [*n*]cumulenes should be less prone deprotonation. In addition, the potential energy profile associated with rotation of the terminal groups in [*n*]cumulenes, from *n* = 3 to 9, has been characterized with high precision. This is of great interest because the potential energy of these systems varies with the dihedral angle between the terminal groups, and this variation depends on the length of the cumulenic chain, where the non-local interaction between the terminal atoms becomes more difficult to capture as the cumulenic chain length increases [91].

Theoretical investigations of bis-functionalized [*n*]cumulenes with N-heterocyclic carbenes (NHCs) have also been carried out, and by calculating hyperhomodesmotic reactions, it has been determined that even [*n*]cumulenes are more stable than odd ones, by up to more than 25 kcal/mol [92]. However, these differences can be dramatically reduced, depending on the type of NHC, but this is consistent with Bestmann's hypothesis, which states that even [*n*]cumulenes are stabilized by *push–push* and odd ones by *push–pull* substitution [93–94]. Although, so far, this hypothesis has only been partially confirmed through the unsuccessful efforts to synthesize bis-functionalized [3]- and [5]cumulenes with NHCs [95–96]. Otherwise, the relative stability of sulfur [*n*]cumulenes (SC*_n_*S) has also been evaluated and by calculating relative energies, it was concluded that structures with an even number of carbon atoms are more stable in the triplet state, while odd ones are more stable in the singlet state; additionally, it was also observed that the proton affinity of these compounds increases as the cumulenic chain increases, indicating that longer chains are more basic [97].

Other theoretical work has been done to study the linearity of these structures that in principle would be expected to be linear, and in general this is true. But it has been found that [4]cumulenes are heavily bent if their terminal functionalizations are poor π-acceptor carbenes [92] or if reduced with cesium to synthesize the corresponding dianion [49]. Moreover, in an early study of [*n*]cumulenones, it was concluded that the barriers to linearity for propadienone (H_2_C_3_O), butatrienone (H_2_C_4_O), and pentatetraenone (H_2_C_5_O) were less than 1 kJ/mol [98]. But, in a later investigation, it was determined that [*n*]cumulenones with more than 3 carbon atoms tend to have angles close to 180°, whereas structures with fewer carbon atoms are bent but very flexible [99]. These findings were consistent with a subsequent study, which reported that the barrier to linearity for propadienone is ≈5 kJ/mol, whereas for butatrienone and pentatetraenone it is <0.5 kJ/mol [100]. This trend toward linearity has been reported for other similar compounds, such as [*n*]cumulenothiones (H_2_C*_n_*S), where chains from 3 to 9 carbon atoms have been evaluated, and it was found that the lowest energy structures are always singlets, essentially linear; thus, they exhibit a more marked tendency toward linearity than [*n*]cumulenonones [101]. Other systems with a behavior closer to [*n*]cumulenones are [*n*]oxycumulenes (OC*_n_*O), since it has been determined that structures with more than 4 carbon atoms are essentially linear, while those with fewer carbon atoms are bent [102].

### Coordination

#### Metalla[*n*]cumulenes

**Figure 7:**
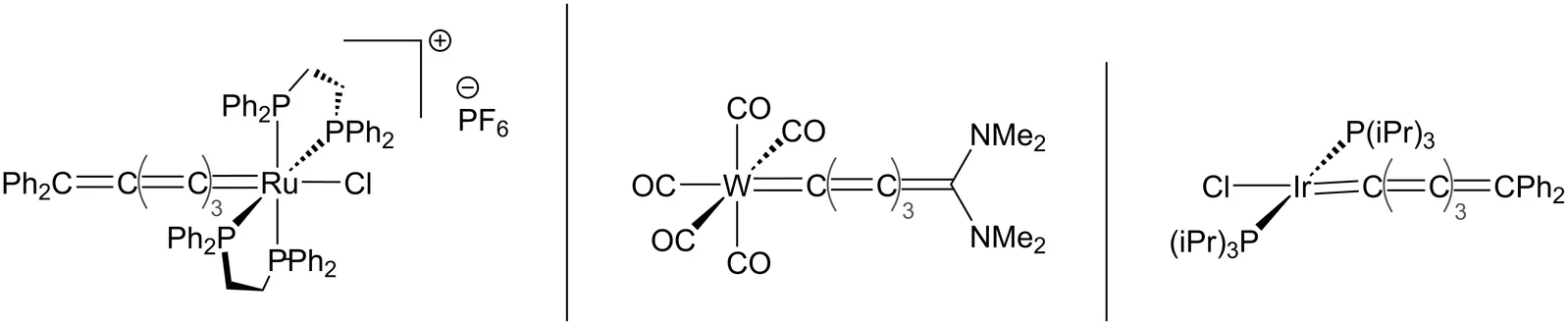
Experimentally characterized metalla[4]cumulenes [107–109].

At this point, we have noted that [*n*]cumulenes can be functionalized with a wide variety of organic groups, but as seen in Figure 7, the terminal sp^2^ carbon atom of the cumulenic chain can be replaced by a double-bonded transition metal fragment (L*_n_*M). These organometallic compounds are known as metallacumulenes (or metalla[*n*]cumulenes, where “*n*” also refers to the number of cumulated C=C double bonds) and arise from the coordination of a metal center with low oxidation state and an [*n*]cumulenylidene derivative. The formation of this M=C double bond consists of three contributions: a σ-donation, from the HOMO–1 of the unsaturated carbene, to the nd_z²_ metal orbital, and two π-backbondings, from the nd_xz_ and nd_yz_ metal orbitals, to the LUMO and LUMO+1 of the unsaturated carbene [103–106].

**Figure 8:**
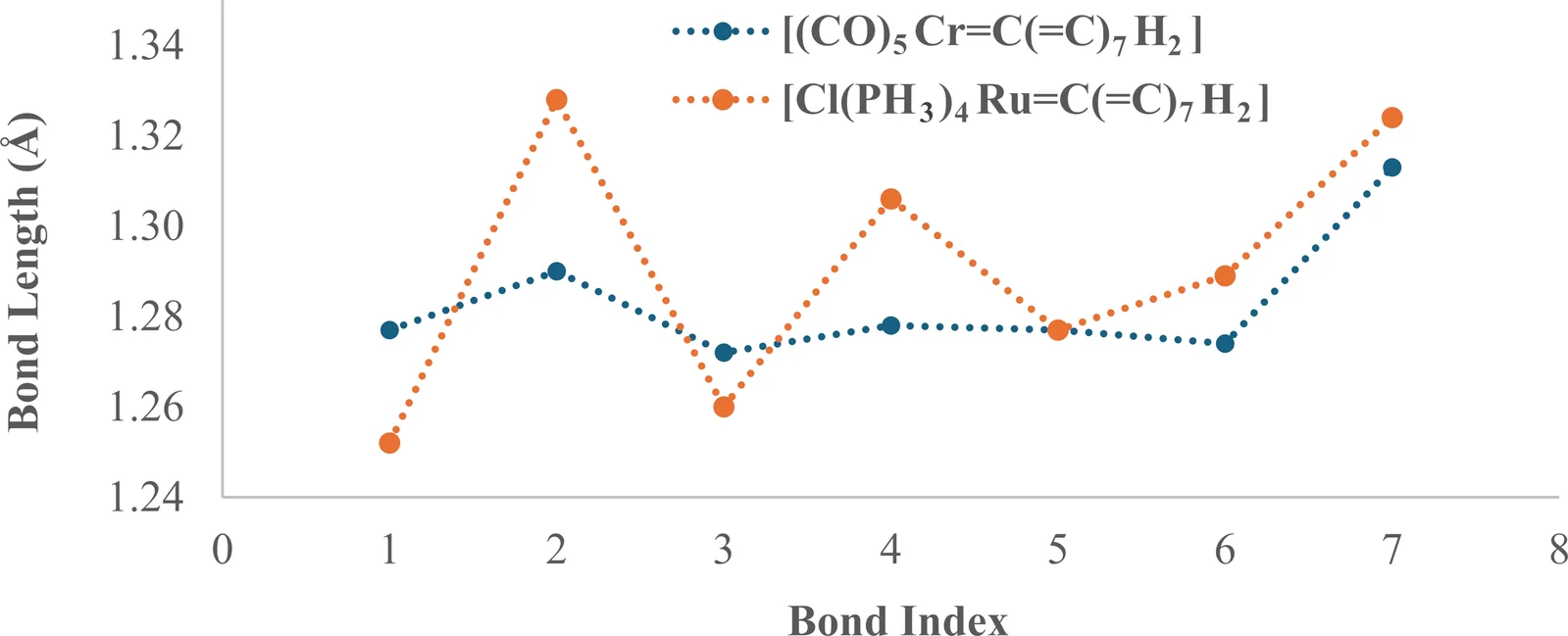
Comparison of the C=C equilibrium bond lengths calculated for [7]cumulenylidene chains with different metal fragments. Theoretical values computed with the (CO)_5_Cr fragment, at the ADF/TZP level of theory, are shown in blue [106], and theoretical values computed with the Cl(PH_3_)_4_Ru fragment, at the ADF/TZ level of theory, are shown in orange [110].

**Figure 9:**
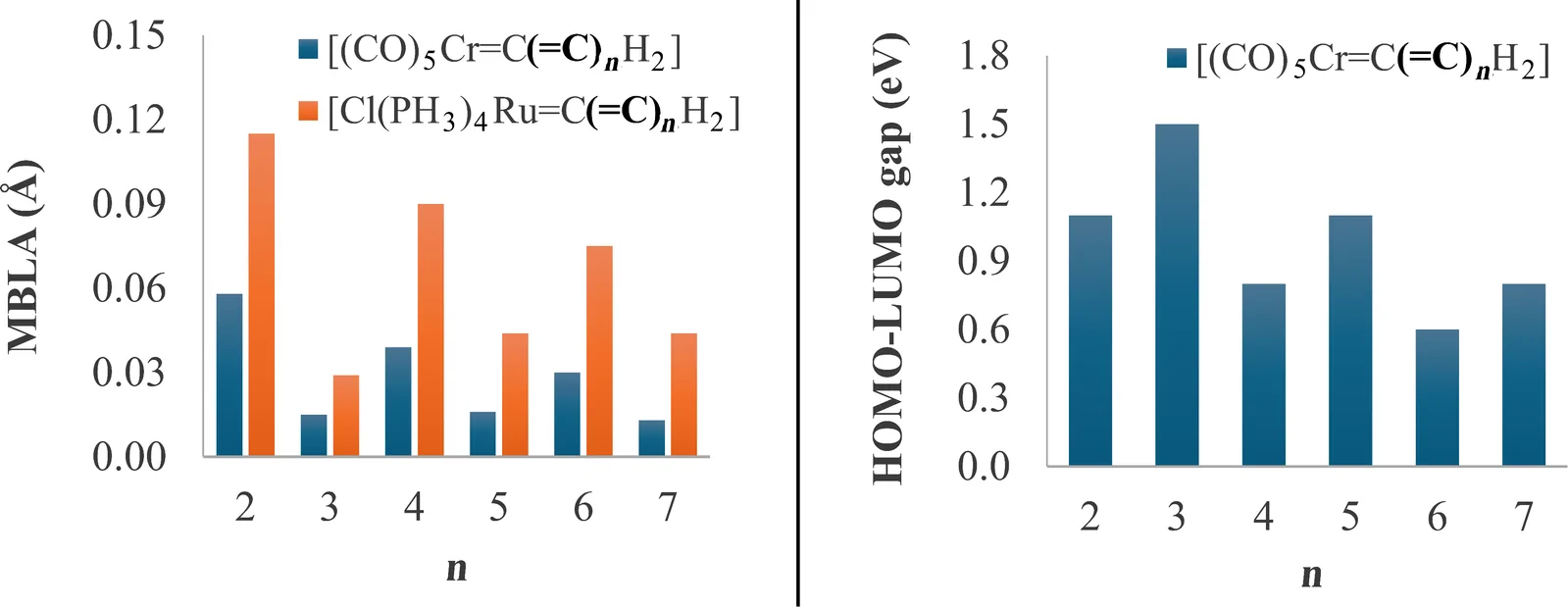
Comparison of the calculated mean bond length alternations (MBLA) and HOMO–LUMO gaps for [*n*]cumulenylidene chains with different metal fragments. Theoretical values computed with the (CO)_5_Cr fragment, at the ADF/TZP level of theory, are shown in blue [106], and theoretical values computed with the Cl(PH_3_)_4_Ru fragment, at the ADF/TZ level of theory, are shown in orange [110].

As expected, in Figure 8 and Figure 9 it can be observed that [*n*]cumulenylidene chains also exhibit BLA and MBLA, and as for [*n*]cumulenes some other trends can be extracted depending on the length and the parity of the chain:

**I. As the [** ***n*** **]cumulenylidene chain increases, the MBLA values decrease (tending to an asymptotic limit).**

As seen in Figure 8, this is because the distances of the C_sp_=C_sp_ double bonds are equalized, although this effect is more evident in [(CO)_5_Cr(=C)_8_H_2_], since the average BLA of its most central (or most internal) pair of C=C double bonds is only 0.004 Å.

**II. Even [** ***n*** **]cumulenylidene chains (** ***n*** **= 2, 4, and 6) exhibit higher MBLA values compared to their odd homologues (** ***n*** **= 3, 5, and 7).**

**Figure 10:**
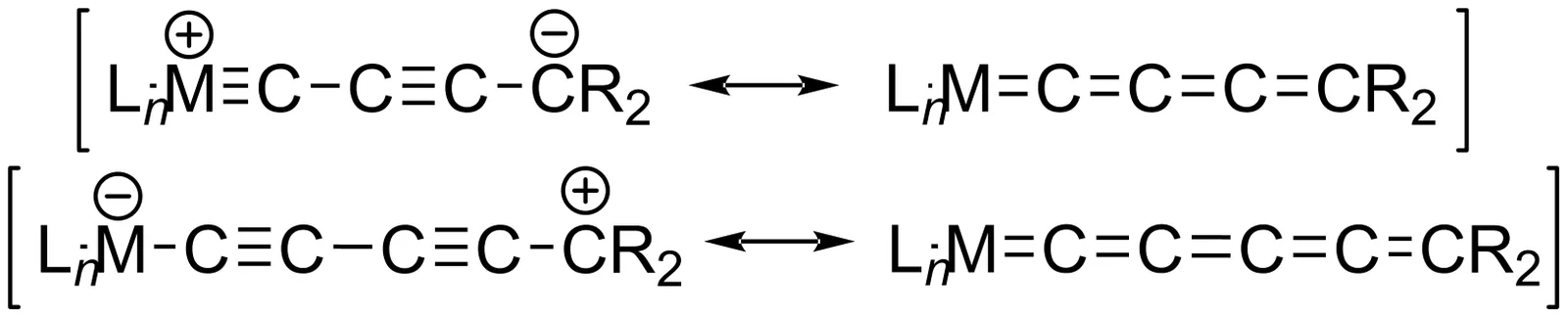
Zwitterionic resonance structures for [3]- and [4]cumulenylidene coordination compounds.

Naturally, this trend can only be appreciated through theoretical studies, since currently, the experimental limit is the metalla[4]cumulenes. Nevertheless, the reason for this trend always depends on the nature of the terminal functionalizations. Through theoretical studies, it has been determined that BLA increases in odd [*n*]cumulenylidene chains when they are functionalized with electron-withdrawing π groups, while electron-donating π groups increase BLA in even ones. This is because, in each case, the zwitterionic resonance shown in Figure 10 is promoted by the presence of the corresponding functional group [103–104].

**III. As the [*n*]cumulenylidene chain increases, the HOMO–LUMO gaps decrease** [103–104,110]**.**

This is because delocalization also increases as the [*n*]cumulenylidene chain length increases and consequently, the HOMO–LUMO gaps decrease.

**IV. Even [** ***n*** **]cumulenylidene chains (** ***n*** **= 2, 4, and 6) exhibit smaller HOMO–LUMO gap values compared to their odd homologues (** ***n*** **= 3, 5, and 7).**

Again, this trend depends on the nature of the terminal functionalizations. Theoretical studies have shown that the HOMO–LUMO gap increases in even [*n*]cumulenylidene chains but decreases in odd ones when they are functionalized with electron-donating π groups, whereas electron-withdrawing π groups produce the opposite effect [103–104]. This occurs because, in even chains, electron-donating π groups slightly increase LUMO's energy but notably decrease HOMO's energy, while, in odd chains, electron-withdrawing π groups slightly decrease HOMO's energy but significantly increase LUMO's energy [104].

Now then, the analysis of FMOs has also allowed to predict the reactivity of higher metalla[*n*]cumulenes (*n* ≥ 4) to electrophilic and nucleophilic attacks. In this way, it has been determined that for d^6^ and d^8^ coordination compounds, the [*n*]cumulenylidene chains have the LUMO located mainly on their odd carbon atoms and the HOMO on their even ones; this determines the regioselectivity of the electrophilic and nucleophilic attack on these atoms, respectively [104–106,110]. On the other hand, the regioselectivity is reversed for d^4^ coordination compounds. While for d^2^ ones, the regioselectivity of the electrophilic and nucleophilic attack is mainly located on the odd carbon atoms [103,105].

It is worth mentioning that, through theoretical calculations, odd metalla[*n*]cumulenes also exhibit electrohelicity, but not much research has really been done in this regard; currently, only a theoretical study has been carried out to quantify the helical morphology of one of these compounds through the MAD index [64]. Another important aspect is that, if the M=C double bond length is considered (≈1.8 Å), consequently metalla[*n*]cumulenes also exhibit reverse BLA.

#### Transition-metal capped carbon chains

**Figure 11:**
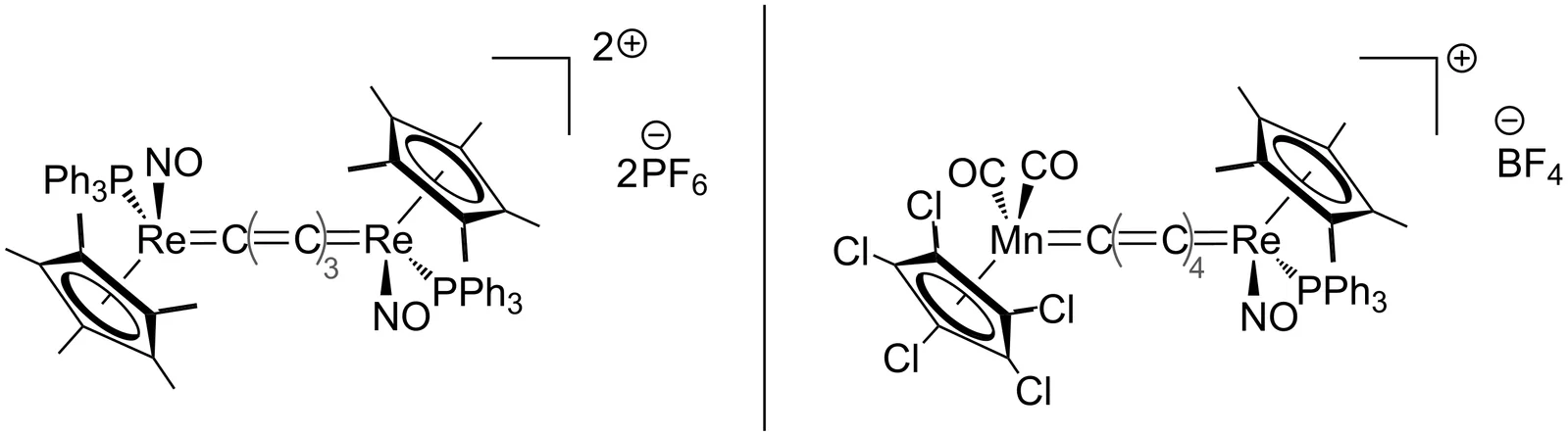
Experimentally characterized cumulenic MCCs [113–114].

So far, we have noted that [*n*]cumulenylidene chains have geometric and electronic properties that correspond to [*n*]cumulenes. But this can change completely with the presence of two transition metal derivatives at the ends. These organometallic compounds are known as transition-metal-capped carbon chains (MCC) [111], and as observed in Figure 11, the pair of metal fragments correspond to half-sandwich compounds of the CpMLL′ type (L, L′ = CO, NO, PR_3_; M = Mn, Re, Fe, Ru) [112]. However, Figure 11 shows only a couple of particular cases, since it′s much more common for the chains to be of a polyynic nature; but, ideally, the presence of a cumulenic structure is sought for the design of molecular wires.

In this regard, through theoretical studies and experimental observations, three factors have been determined that promote the presence of cumulenic chains in these compounds:

1. **Odd number of carbon atoms:** chains with 5, 7, and 9 carbon atoms exhibit a considerable cumulenic character because they have significantly lower MBLA values [111,115].
2. **Equivalent metal fragments:** heterobimetallic MCCs usually exhibit polyynic chains due to the difference in electronegativities between the metal centers [112,116].
3. **Dicationic nature:** It has been experimentally observed that neutral MCCs exhibit polyynic chains, while the removal of two electrons reinforces the cumulenic character [111,115].

Through theoretical research, it has also been shown that MCCs of cumulenic nature are distinguished when their chains exhibit an even or odd number of carbon atoms; the study interval covers from one to 25 carbon atoms, and these differences are listed below [111,115]:

- Odd carbon chains present non-degenerate FMOs, while even carbon chains present quasi-degenerate FMOs.
- Even carbon chains exhibit smaller HOMO–LUMO gaps compared to their odd homologues.
- Odd carbon chains have lower MBLA values compared to their even homologues.
- The closed-shell singlet electronic state is preferred in odd carbon chains, while even carbon chains exhibit closed-shell singlet and triplet states very close in energy.

In addition, as the cumulenic chain length increases, the singlet-triplet states, the HOMO–LUMO gaps and the MBLA values show a similar convergence [111,115].

**Figure 12:**
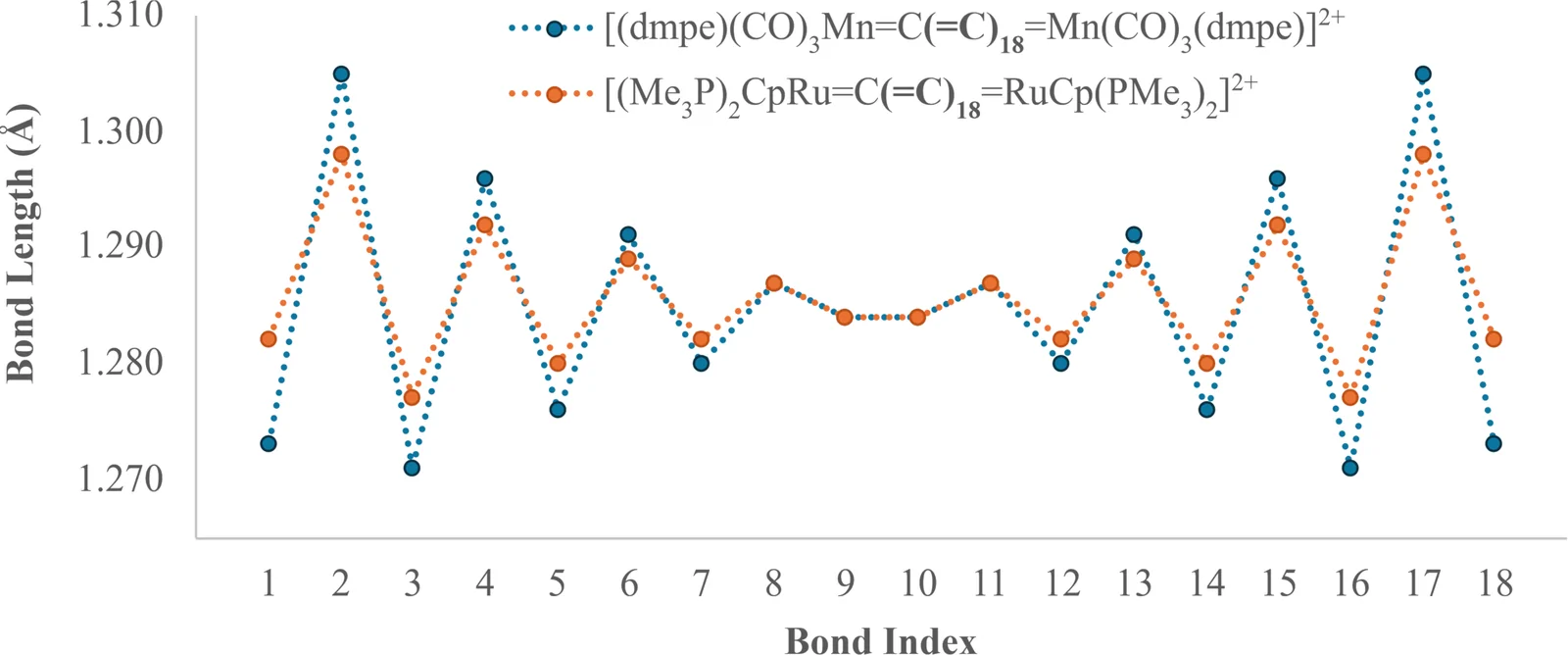
Comparison of the C=C equilibrium bond lengths calculated for different cumulenic MCCs, in a closed-shell singlet electronic state, at the B3LYP-D3(BJ)/def2-SVP level of theory [111].

It is worth noting that, through theoretical calculations, cumulenic MCCs also exhibit electrohelicity, but this has not been investigated further [111]. Another important aspect is that, if the M=C double bond length is considered (≈1.8 Å), consequently cumulenic MCCs also exhibit reverse BLA (Figure 12).

#### Metal–[*n*]cumulene coordination compounds

**Figure 13:**
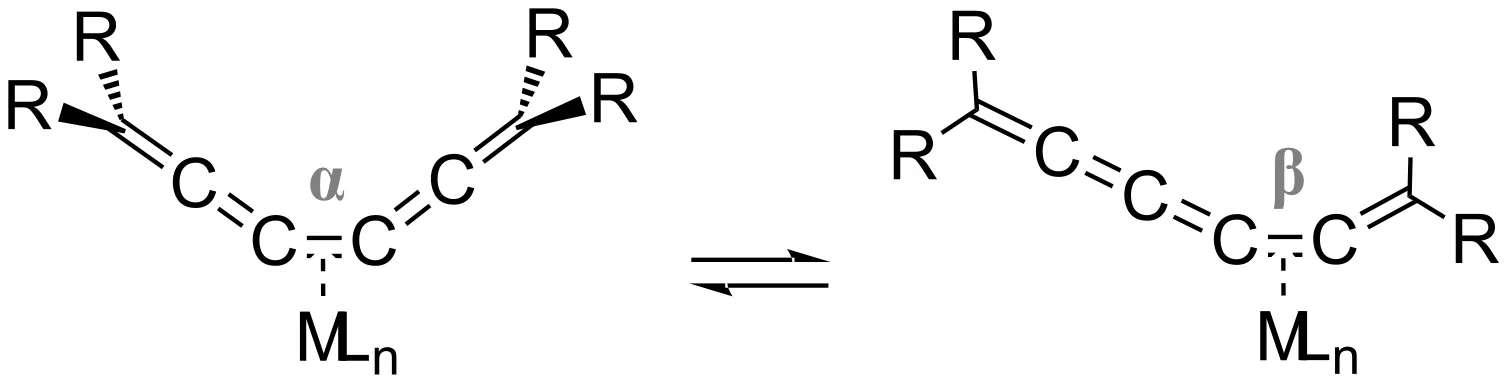
Haptotropic shift in metal-[5]cumulene coordination compounds.

Now then, as observed in Figure 13, [*n*]cumulenes can also act as η^2^-ligands. The formation of this coordination bond essentially consists of two contributions: a σ-donation of one of the π bonds of the [*n*]cumulene to one of the empty nd orbitals of the metal center (with low oxidation state) and the subsequent π-backbonding from a filled nd orbital to the empty π* orbital. This coordination mode gives rise to haptotropic shifts, indicating that the metal fragment can move across the double bonds of the cumulenic chain [117–121].

In this regard, controlling the reversibility of haptotropic shifts in favor of a tautomer of interest may be especially useful for the design of molecular switches. However, to date, no external stimulus capable of achieving this behavior has been discovered in this class of compounds. But through theoretical studies, it has been determined that the steric bulk of the terminal functionalizations in [5]cumulenes plays a fundamental role in tautomeric preferences. Specifically, the presence of bulky substituents, such as *tert*-butyl groups, promotes the formation of symmetric coordination compounds (α), while less bulky functional groups, such as phenyls, favor the formation of asymmetric coordination compounds (β) [117].

#### Magnetic properties

Single-molecule magnets (SMMs) are organometallic compounds that exhibit superparamagnetic properties, such as high magnetic anisotropy and conservation of their magnetization for a relatively long time, in the absence of an applied magnetic field and at low temperatures. Therefore, they have been considered as elements for the development of memory storage nanodevices and for the construction of quantum computers. In general, the design of SMMs involves the presence of transition metals, lanthanides, or actinides bound to highly conjugated and delocalized molecules, which contribute to the increase in the magnetic moment and promote spin delocalization [122–123].

In this regard, theoretical studies have suggested that [*n*]cumulenes may provide an optimal route for spin delocalization thanks to their rigid and continuous arrangement of cumulated π bonds, contributing to an increase in magnetic exchange coupling. Furthermore, theoretical investigations have also suggested that the diradical character and a convenient structural design, could provide a precise control of the magnetic properties, by modulating the interaction between the unpaired electron pair, giving rise to a singlet (antiferromagnetic) or triplet (ferromagnetic) ground state. However, although this pair of hypotheses hasn't yet been experimentally confirmed, the syntheses of bis-functionalized [3]- and [5]cumulenes with phenalenyls are expected to produce promising prospects for SMMs [124–125]. On the other hand, this pair of hypotheses has already been studied theoretically, through [*n*]cumulenes disubstituted at opposite ends with the nitronyl nitroxide radical (NN), which produce ferromagnetic ground states in even [*n*]cumulenes and antiferromagnetic in odd ones. This agrees with the spin density alternation rule, based on Hund's rule. This rule states that the spin density alternates along the conjugated bonds of a molecule. Although in this case we have cumulated bonds, the principle is preserved. When two spin centers are connected by an even number of cumulated bonds, the spin densities at the ends align in the same direction, causing a ferromagnetic interaction and, consequently, a positive magnetic exchange coupling constant (*J*). In contrast, when two spin centers are connected by an odd number of cumulated bonds, the spin densities at the ends align in opposite directions, resulting in an antiferromagnetic interaction with a negative *J* [71,126]. Furthermore, it was observed that there was an increase in the spin density and in the magnitude of the *J* constant as the cumulenic chain length increased. But odd [*n*]cumulenes exhibit higher magnitudes in the *J* constant compared to their even homologues. This is because the π interaction between the cumulenic chain and the NN groups decreases notably when the terminal functionalizations aren't in the same plane, causing a small magnitude of the *J* constant [71].

Subsequently, di-substitution at opposite ends was studied with metallocenes; specifically, cobaltocene [20], chromocene, nickelocene and the mixed chromocene-nickelocene configuration [21]. These terminal functionalizations produce ferromagnetic ground states in homobimetallic derivatives of even [*n*]cumulenes and antiferromagnetic in odd ones [20–21], but the trend is reversed in heterobimetallic derivatives [21]. Furthermore, it was determined that the spin density and the *J* constant increase as the cumulenic length chain increases, but only for derivatives of chromocene, nickelocene and their mixed configuration [21]; while for cobaltocene derivatives, the *J* constant increases as the cumulenic chain length increases, but the spin density decreases [20]. In all cases, a small magnitude of the coupling constant was also observed for even [*n*]cumulenes [20–21], reaffirming that the π interaction between the cumulenic chain and the terminal functionalizations decreases notably when the terminal functionalizations aren't in the same plane. Moreover, all derivatives were subjected to magnetic anisotropy studies, but only the nickelocene and cobaltocene systems exhibited negative zero-field splitting parameters (D); therefore, it was concluded that only these systems are promising prospects for SMMs [20–21].

SMMs also have potential applications in spintronics as spin filters. In this regard, through theoretical studies, it has been investigated that cobaltocene-monofunctionalized [*n*]cumulenes can generate a spin-polarized electron current, demonstrating a clear separation in their spin-up (↑) and spin-down (↓) transmission curves. But odd [*n*]cumulenes produce a more notable separation, thanks to their smaller HOMO–LUMO gaps; therefore, they are more efficient as spin filters [20].

#### Singlet fission

Through theoretical studies, it has been proposed that the diradical character could be a prerequisite for the design of chromophores with nonlinear optical properties, such as two-photon absorption and singlet fission [127–128]. Particularly, singlet fission is a potentially useful photophysical process for the production of organic photovoltaic (OPV) cells, since it involves the radiationless conversion of one singlet exciton (S_1_) into two triplet excitons (T_1_), via an intermediate spin-correlated triplet-pair state. The efficiency of this process increases when it is exoergic, since it maximizes the production of triplets and the diradical character contributes to stabilizing these states, which is beneficial to increasing the photoelectric conversion and ideally, would raise the maximum thermodynamic efficiency far beyond the Shockley–Queisser limit [129–131].

Recently, theoretical studies have suggested that [*n*]cumulenes could carry out this process through terminal functionalizations that allow increasing and stabilizing their diradical character, such as carbenes, since it has been shown that in general, they have the capacity to stabilize radicals [131–132]. This promising behavior was initially studied by bis-functionalizing [3]- and [5]cumulenes with cyclic (alkyl)(amino)carbenes (CAACs), but none of these compounds turned out to be good candidates for singlet fission chromophores, mainly because the structural rearrangements associated with S_0_ → S_1_ and S_0_ → T_1_ excitations lead to very high kinetic barriers [127]. Otherwise, there is also a wide variety of carbenes that have also been studied for this purpose, such as mesoionics (MIC), pyrazolinylidenes (pyz), cyclic bent allenes (CBA), diphenylcarbene (Ph_2_C), carbodicarbenes (CDC) and NHCs. Through these studies, it has been determined that CAAC, MIC and NHCs decrease the diradical character in even and odd [*n*]cumulenes, while CDC and CBA lead to an open-shell ground state. Although CDC has been predicted to be better at stabilizing organic radicals. Moreover, interestingly, 1,1,6,6-tetraphenyl-[5]cumulene has also been determined to be a promising candidate for singlet fission, as it satisfies the energy matching condition for an exoergic process: 2·*E*(T_1_) ≈ *E*(S_1_) and also satisfies the condition for avoiding the recombination of triplet excitons: 2·*E*(T_1_) < *E*(T_2_) [128].

## Conclusion and Perspective

Historically, [*n*]cumulenes have occupied a niche area of organic chemistry for just over a century [133]. Only in recent years, however, have they evolved from “exotic” unsaturated chains into highly tunable platforms with potential applications in molecular electronics and materials science [11–16]. Notably, most of these advances have been achieved only through odd [*n*]cumulenes, whereas experimental studies on even [*n*]cumulenes remain comparatively scarce. Since the first synthesis of [4]cumulene derivatives [134] to date, only 34 such compounds have been reported, of which just 21 have been isolated as solids [25].

Here, we emphasize possible applications and properties of [*n*]cumulenes, most of these based on theoretical predictions or from theoretical design principles. We also showed that even [*n*]cumulenes are often less compatible with many of these applications. Nevertheless, a central message that emerges from this review is the critical role of terminal functionalizations in unlocking their potential. Appropriate substitution can suppress the simultaneous presence of both helicities in their electronic structure, thereby enhancing their suitability for applications such as optical detection, photoanalysis, enantioselective transformations, screw electromagnetism, molecular or anti-ohmic wires, and systems capable of inducing substantial magnetic fields [24,60,79].

Closing the current gap between theoretical predictions and experimental validation should define the next stage of the field, enabling components based on [*n*]cumulenes, whose optical, electronic, and magnetic behaviors can be precisely programmed at the molecular level. Future methodological advances that stabilize longer even [*n*]cumulenes (*n* > 4) – or generate them under mild and controllable conditions – will be crucial for conducting direct experimental tests of predictions that are currently based solely on theoretical studies. In this regard, it would be interesting to confirm Bestmann's hypothesis, which states that even [*n*]cumulenes are stabilized by *push–push* substitution [93–94]. This has already been confirmed theoretically with NHCs [92], and inorganic hetero-[4]cumulenes inspired by this approach have already been synthesized [135].

## Data Availability

Data sharing is not applicable, as no new data were generated in this review.
